# Application of an Integrative Computational Framework in Trancriptomic Data of Atherosclerotic Mice Suggests Numerous Molecular Players

**DOI:** 10.1155/2012/453513

**Published:** 2012-11-06

**Authors:** Olga Papadodima, Allan Sirsjö, Fragiskos N. Kolisis, Aristotelis Chatziioannou

**Affiliations:** ^1^Metabolic Engineering and Bioinformatics Program, Institute of Biological Research and Biotechnology, National Hellenic Research Foundation, 48 Vas. Constantinou Avenue, 11635 Athens, Greece; ^2^Division of Clinical Medicine, School of Health and Medical Sciences, Örebro University, Örebro SE-701 82, Sweden; ^3^Biotechnology Laboratory, School of Chemical Engineering, Zografou Campus, National Technical University of Athens, 15780 Athens, Greece

## Abstract

Atherosclerosis is a multifactorial disease involving a lot of genes and proteins recruited throughout its manifestation. The present study aims to exploit bioinformatic tools in order to analyze microarray data of atherosclerotic aortic lesions of ApoE knockout mice, a model widely used in atherosclerosis research. In particular, a dynamic analysis was performed among young and aged animals, resulting in a list of 852 significantly altered genes. Pathway analysis indicated alterations in critical cellular processes related to cell communication and signal transduction, immune response, lipid transport, and metabolism. Cluster analysis partitioned the significantly differentiated genes in three major clusters of similar expression profile. Promoter analysis applied to functional related groups of the same cluster revealed shared putative *cis*-elements potentially contributing to a common regulatory mechanism. Finally, by reverse engineering the functional relevance of differentially expressed genes with specific cellular pathways, putative genes acting as hubs, were identified, linking functionally disparate cellular processes in the context of traditional molecular description.

## 1. Introduction

Atherosclerosis is the leading pathological contributor to cardiovascular morbidity and mortality worldwide, characterized by the progressive accumulation of lipid and fibrous depositions in the vessel wall of medium-sized and large arteries. Although it has traditionally been viewed as simple deposition of lipids within the vessel wall, it is now assumed that atherosclerosis is a multifactorial disease that involves several genes and proteins, activated during its genesis, progress, and phenotypic manifestation. During atherogenesis, a complex endothelial activation and dysfunction induced by elevated and modified low-density lipoproteins and many other factors leads to a compensatory inflammatory response [1]. Current evidence supports a central role for inflammation, in all phases of the atherosclerotic process. Substantial biological data implicate inflammatory pathways in early atherogenesis, in the progression of lesions, and finally in the thrombotic complications of this disease [2]. 

Clinical investigations, population studies, and cell culture experiments have provided important clues to the pathogenesis of atherosclerosis. However, the use of animal models has had a crucial contribution in the research of the atherosclerotic course. Atherosclerosis will not be developed in laboratory mice under normal conditions. However, targeted deletion of the gene for Apolipoprotein E (ApoE knockout mice) leads to severe hypercholesterolemia and spontaneous atherosclerosis [3]. For this reason, ApoE deficient mice are widely used to study atherosclerosis [4]. ApoE is a ligand for receptors that clear chylomicrons and very low-density lipoprotein remnants. Furthermore, a number of population studies suggest that ApoE genotype predicts the risk of developing atherosclerosis and related diseases [5]. 

**Figure 1:**
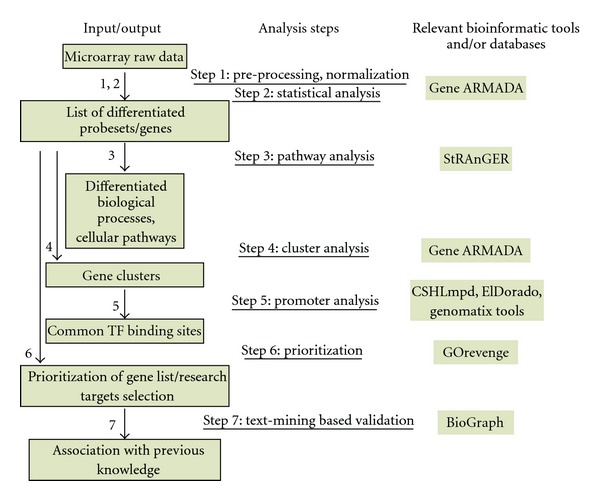
Schematic representation of the proposed workflow. Arrows depict the various analysis steps. The bioinformatic tools and databases used for the implementation of each step are also shown.

In this study, we propose a framework for efficient translational bioinformatic analysis showcased on a microarray dataset concerning biological specimen from ApoE knockout mice. Gene expression data of wild type and ApoE knockout 6-, 32-, and 78-week-old mice have been utilized. This dataset was previously presented in a detailed work studying atherosclerosis and inflammatory pathways during aging [6]. The proposed workflow comprises seven basic steps (Figure 1): raw data pre-processing and normalization, statistical selection, pathway analysis, clustering, promoter analysis, gene list prioritization, exploiting network centrality criteria enabling identification of interesting research targets, and finally intelligent text-mining based validation of the selected molecular targets from the broader biomedical literature. Aim here is to combine several bioinformatic tools, in a unique, generic, computational workflow, appropriate for batch processing, able to confer reliable functional knowledge, regarding different aspects of the biological mechanism investigated, in order to highlight critical, underlying, molecular determinants governing it. Besides an algorithmic proposition, the workflow presented here is currently in the phase of implementation, regarding the seamless integration of its constituent modules, exploiting the web service technology [6], accessible through a user-friendly web application, and enabling automated extraction of consolidated biological knowledge, in the form of concrete functional scenario, from high-volume data omic datasets. Nowadays, several tools are available for the implementation of each analysis step. For instance, Bioconductor [7] represents one of the richest repositories of statistical algorithms and has become, by all means, a standard for microarray data analysis but its command line interface limits its usability to many, wet-lab oriented, biological experts. To overcome this limitation, user friendly software packages for normalization, statistical analysis, and visualization of microarray expression data have been developed, like Gene ARMADA [8] and FlexArray [9]. Regarding pathway analysis, multiple software tools are exploiting ontological vocabularies to target the issue of detecting over-represented terms in microarray datasets, aiming to indicate possibly altered molecular processes [10–14]. Regarding promoter analysis, it remains one of the most intricate issues regarding the efficient mining of gene lists derived from transcriptomicexperiments. Different promoter sequence databases [15, 16] and bioinformatic tools [17, 18] have been developed but still elucidation of gene transcription regulating networks remains a great challenge. The derivation of functional information regarding gene function, exploiting semantic similarity criteria, represents a promising, yet fuzzy and increasingly bewildering in its interpretation, approach. Several criteria and measures have been proposed [19], however it is GOrevenge [20], which instead of focusing in the neighboring genes, it highlights linker genes, associated with discrete cellular functions (distant in terms of semantic similarity). Finally BioGraph [21] is a data integration and data mining platform for the exploration and discovery of biomedical information. The platform offers prioritizations of putative disease genes, supported by functional hypotheses. BioGraph can retrospectively confirm recently discovered disease genes and identify potential susceptibility genes, without requiring prior domain knowledge, outperforming from other text-mining applications in the field of biomedicine. In the present analysis we show that integration of different analysis snapshots, as obtained through bioinformatic analyses, results in reliable, prioritized and informative lists of differentiated genes, and/or molecular pathways. 

## 2. Materials and Methods

### 2.1. Microarray Data

The mouse dataset used is the GSE 10000, available at Gene Expression Omnibus (GEO) database. Microarrays were prepared following MIAME guidelines, as described in [22]. Briefly, RNA from aortic tissue of ApoE knockout and wild type animals was hybridized on Affymetrix 430 2.0 Arrays. Three different ages were studied: 6, 32, and 78 weeks.

### 2.2. Microarray Data Analysis and Statistical Analysis

Microarray data analysis was performed in Gene ARMADA [8]. Briefly, background correction was performed employing its gcRMA method followed by Quantile Normalization. Data were log2 transformed to comply with the normality assumption. Differentially expressed genes in at least one among all the experimental conditions were identified using Gene ARMADA, by performing 1-way ANOVA on log2 transformed fold changes. The resulting gene list was obtained by setting the *P* value threshold to 0.01, the False Discovery Rate (FDR) threshold to 0.05 and by removing genes that presented a fold change below *|*1*|*, in log2 scale, in all conditions. 

### 2.3. Prioritized Pathway/Functional Analysis

Statistical enrichment analysis was performed using StRAnGER [8], in order to highlight biological processes including statistically significant numbers of the ANOVA derived genes. In order to expand our knowledge regarding the functional implication of genes in various cellular processes, prioritizing them according to their centrality, we used the online tool GOrevenge [20] with the following settings: Aspect: BP (Biological Process), Distance: Resnik, Algorithm: BubbleGene, and Relaxation: 0.15.

### 2.4. Cluster and Promoter Analysis

The list derived from ANOVA was subjected to hierarchical clustering (linkage method: Average, distance: Cosine) in Gene ARMADA. Promoter sequences from −700 to +300, relative to transcription start site, were downloaded for mouse and human from Cold Spring Harbor Laboratory Mammalian Promoter Database (CSHLmpd) [16]. In the cases that alternative promoters were given for the same gene, we selected the one defined as the “best" at [16]. For promoters that we could not detect in this database, we additionally searched the ElDorado database [15]. In the case of genes with multiple promoters supported by different transcripts, we selected the one corresponding to the Reference Sequence of NCBI. To analyze each promoter set for common TF binding sites, we used the MatInspector software [18]. The parameters used were as follows: Library version: Matrix Library 8.0, Matrix group: Vertebrates, Transcription Factor sites common to: 85% of input sequences, Core similarity: 0.75, Matrix similarity: Optimized, and *P* value cut-off was set at 0.01. Among the identified TF sites only those that were present in both species were considered. 

## 3. Results 

### 3.1. Statistically Significant Differentiated Genes

**Figure 2:**
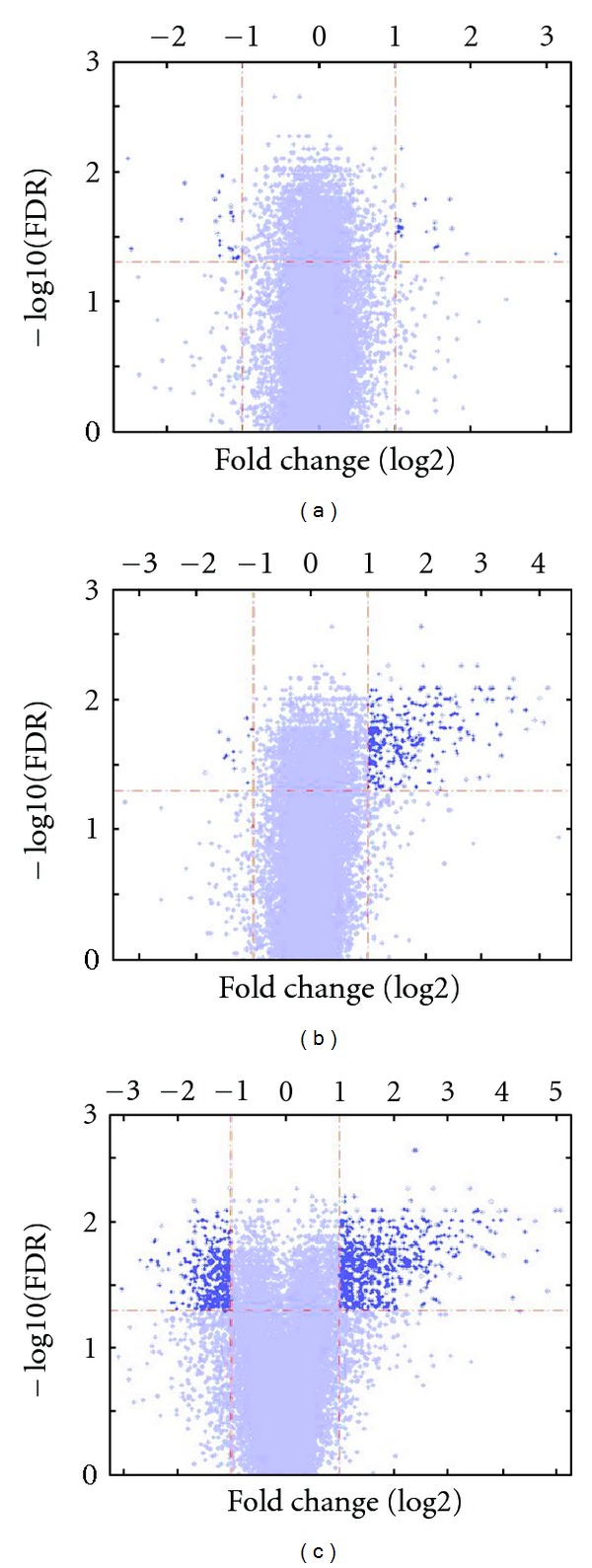
Volcano plots of the gene list as yielded by ANOVA. Each panel represents filtered and normalized data from each experimental condition (3, 6, and 78 weeks old mice). The horizontal axes depict the fold change ratio between ApoE deficient and wild type mice, for each age in log2 scale, while the vertical axes represent statistical significance by depicting the −log10 (FDR).

To obtain the aortic gene expression profile of ApoE deficient mice in 6-, 32-, and 78-week-old mice we analyzed the GSE 10000 dataset, containing expression data of aortic tissue from wild type and ApoE knockout mice. Specifically, in order to identify significant alterations among all three tested ages, 1-way ANOVA was applied to expression fold changes between expression in ApoE knockout and wild type animals (*P* value <0.01 and FDR <0.05) coupled with further filtering on fold change (>*|*1*|* in at least one condition in log2 scale). A list of 1033 significantly differentiated probesets was obtained (Supplementary Table 1; see supplementary material available online at doi:10.1155/2012/453513), depicted per time point using a volcano plot representation (Figure 2). These 1033 probesets correspond to 852 annotated genes. It is characteristic that in 6 weeks old mice the number of significantly altered genes is very limited, in 32 weeks old mice the majority of differentiated genes are upregulated, while in 78 weeks old mice we have the greater number of differentiated genes.

### 3.2. Pathway Analysis

For the scope of gaining further insight concerning the biological functionalities of gene expression alterations in a more systematic way, the list of 852 significantly differentiated genes yielded from ANOVA was subjected to statistical enrichment analysis using StRAnGER, exploiting GO terms and Kegg pathways for the task of the functional annotation of the interrogated genes. GO-based analysis, focused on the categories of “Biological Process” with a hypergeometric *P* value <0.001, suggested several processes as possibly differentiated, which are presented in Table 1. A lot of central molecular mechanisms emerge as altered, as indicated by the GO categories listed in Table 1, like differentiation, proliferation (inferred by cell cycle and cell division GO terms), apoptosis, cell adhesion, signal transduction, and immune response. Kegg pathway-based analysis also indicates alterations in cytokine signaling, cell adhesion, and signal transduction (Supplementary Table 2). It is important to note that in conformity to the well established relationship of atherosclerosis and inflammation, the majority (29 out of 32) of the genes under the category “immune response” are upregulated suggesting a stimulation of the immunological mechanisms (Table 2).

### 3.3. Cluster Analysis

**Figure 3:**
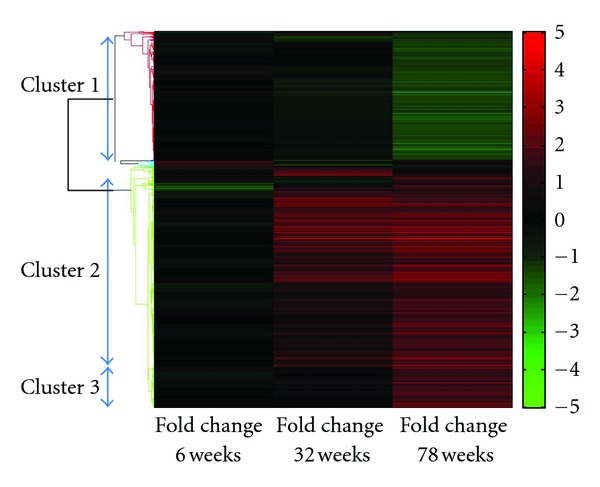
Hierarchical clustering of the 1033 statistically significant differentiated probesets. Fold changes between the gene expressions in ApoE knockout as compared to age-matched wild type mice are grouped in three major clusters.

In order to identify groups of genes presenting similar expression and possibly comprising regulated “waves” of transcription, the list of 1033 significantly differentiated probesets was subjected to hierarchical clustering (Figure 3). Three major clusters can be distinguished: the first one (323 probesets) contains transcripts downregulated in 78 week old mice, while their expression remains close to the control (wild type) level at 6 and 32 weeks. The second cluster (526 probesets) groups genes which are upregulated at 32 weeks and their expression at ApoE knockout mice remains at high levels, as compared to wild type, also at 78 weeks. The third cluster (110 probesets) groups genes whose expression is late upregulated at 78 week old ApoE knockout, as compared to age-matched wild type mice.

Based on these three major clusters, we performed GO-analysis to the genes of each cluster separately. Genes under cluster 1 are functionally connected to processes involved in cell differentiation, adhesion, and signal transduction. Cluster 2 contains the greatest number of genes, which are related mainly to mechanisms involved in immune and inflammatory response as well as lipid metabolism. These processes emerge as significantly altered specifically in the case of cluster 2. Cluster 2 genes are also connected to key cellular processes like signal transduction, apoptosis, cell cycle, and differentiation. Cluster 3 genes are mainly related to mechanisms concerning gene transcription. 

### 3.4. Promoter Analysis

Next, we focused our analysis on small groups of genes presenting similar expression profile, as indicated by cluster analysis, and also being functional relevant, as suggested by GO analysis. In order to investigate whether there are common regulatory transcriptional mechanisms in such groups of genes, we performed a representative promoter analysis in genes of cluster 2 belonging to the GO category of “immune response” either “inflammatory response.” We selected these categories because they appear as significantly altered, scoring at the top of GO analysis prioritization list. We combined the genes of these two groups, resulting at a total number of 36 genes in both categories, because they are functionally relevant, as they represent genes involved in immunological mechanisms. In order to find common putative transcription factor (TF) binding sites in at least a subset of this group, proximal promoter sequences from both mouse and human genomes were extracted from available databases and analyzed as described in Methods. Only common TF binding sites among the two species were considered.  Table 3 summarizes statistically significant TF motif families common in at least 80% of promoter sequences, sorted in descending order in terms of statistical significance. The *P* values, representing the probability to obtain a greater or equal number of sequences with a match in a random sample of the same size as the input sequence set, are precalculated for each binding site and depend on its definition. 

We then examined whether among the significantly differentiated genes we could identify TFs possibly recognizing binding sites presented at Table 3 and thus being involved in the regulation of the relevant genes. Interestingly, among the upregulated genes there are Klf4 and Irf8 TFs, whose binding sites are found at 100% and 86% of the tested promoters, respectively. In particular, Klf4 shows an increased expression at ApoE knockout mice as compared to age-matched wild type both at 36 and 78 weeks (0.62 and 1 fold increase, in log2 scale, resp.) while in 6 weeks the expression of KLf4 is moderately decreased as compared to wild type (−0.46, log2 scale). The expression of Irf8 at ApoE knockout mice is significantly increased both at 36 and 78 weeks (1.9 and 2.61 fold increase, in log2 scale, resp.), while at 6 weeks it remains at the wild type levels. Thus the upregulation of these factors could partially account for the observed upregulation of the immune-related group of genes. 

### 3.5. Identification of Candidate Hub-Genes

In order to expand our knowledge regarding which genes have critical role, taking into consideration their centrality as described in the GO tree, we used the online tool GOrevenge [20]. The list of 852 differentiated genes was submitted to GOrevenge and the analysis was performed based on GO annotations for *Mus musculus* as described in materials and methods section. The derived list of genes, containing candidate linker genes, that is genes participating in many different cellular processes, was partitioned to include only the genes that have been also identified, as statistically significantly differentiated. The derived list (Table 4) contains genes that were identified as significant both by ANOVA and by GOrevenge analysis. The list of genes is prioritized according to the centrality of each gene, as it is reflected by the number of GO biological processes related terms remaining after GOrevenge pruning [20]. Significant molecules involved in signaling and developmental mechanisms emerge as central players. In order to evaluate the relation of these genes with atherosclerosis, which is a principle phenotypic characteristic of ApoE knockout mice, we used the BioGraph platform [21] which utilizes data mining algorithms that exploit textual terms to build a network of heterogeneous relations which link genes with a specific concept (such as genes, proteins, diseases as described in [21]). The resultant BioGraph network describes associations in *Homo sapiens.* By setting atherosclerosis as concept, the relation of each gene with atherosclerosis was assessed and the top 20 genes obtained by GOrevenge were prioritized as shown in Table 5 by BioGraph algorithm. The genes are prioritized according to their score which is a statistical enrichment measure of the relevance of each gene with the inquired context (here specified as atherosclerosis) to the total relations (references) of the gene in the universe of terms. In this way, the user can derive which of its genes are already associated and in what extent with a given disease or generally biological term, and which of them represent novel findings with respect to the investigated pathological phenotype. Since the list of gene symbols used as input to BioGraph represent *Mus musculus* genes, while BioGraph refers to *Homo sapiens* genes, some of them could have different symbol in each species. In the case that a gene symbol was not recognized by BioGraph, we searched the NCBI HomoloGene database [23] in order to find the homologous gene in *Homo sapiens* (e.g., FOXF1 of Table 5 corresponds to Foxf1a of Table 4). 

## 4. Discussion

In this study, we presented a detailed, multi-stage, translational bioinformatic analysis of ApoE knockout mice, exploiting different methods in order to identify critical altered molecular mechanisms and important central players. Our approach was to apply a generic computational framework, which exploits rigorous statistical or computational measures at every analytical step, for the efficient systems level interpretation of the results of ApoE dataset. The workflow proposed here integrates various software products, in a unified translational pipeline, able to cope with versatile, high-volume investigation tasks, and at the same time provide a reliable systemic interpretation for the biological mechanism studied. In this way, a powerful translational backbone is set, which connects the wet-lab part with the theoretical knowledge for the biological problem interrogated, as rescued in molecular databases, controlled ontological vocabularies or the literature. The workflow presented in this study, currently in the phase of implementation as regards to its software components integration, represents an efficient and highly innovative effort, either in terms of speed of analytical performance, as well as real biological value of the results. This is so because it provides results which are qualified from a composite framework that combines ideally both individual and group quality measures, together with an insightful comprehension of the underlying topological networks, actively involved in the mechanism studied. The correlation of the results of the molecular analysis with literature-derived associations manages to highlight and propose promising, novel candidates that have not been studied in the context of the given pathology. They could thus represent ideal targets for further biological experimentation. Maximizing the total information gain encompassing all analytical steps of the proposed workflow represents a critical parameter regarding the implementation of the web application. However as the derivation of automated statistical thresholds for such high-volume data processing in an unsupervised manner both in terms of performance and computational speed is a very challenging task, this still remains an open issue for extensive research work and testing, representing an important point for future work.

Computational technologies are complementary to conventional “wet lab” gene discovery technologies in that they can support the prioritization and comprehension of high-volume molecular data (i.e., omic datasets from a microarray or novel sequencing technologies, associated regions from genome wide association or linkage studies) enabling the efficient selection of the top candidates, under a range of criteria, for further study. In recent years, there are popular tools and environments in the scientific computing realm (data-mining, artificial intelligence, hyper-computing), like the Taverna workflow manager [24] or the RapidMiner solution, formerly known as YALE [25], which enable efficient workflow integration and deployment, exploiting versatile web service repositories, containing hundreds of operators implementing various analytical tasks. Especially Taverna workflow manager, through myExperiment (http://www.myexperiment.org) [26] and Biocatalogue (http://www.biocatalogue.org) [27], integrates an impressive number of workflows and web services all accessible through Taverna, for a very wide range of disparate bioinformatics tasks. However, to the best of our knowledge, the workflow showcased in this work addresses in a sequential, unsupervised fashion disparate tasks enabling and empowering decisively the translational procedure, in a completely innovative yet efficient way.

Applying the proposed workflow to a dataset from ApoE knockout and wild type mice, it was shown that the gene expression profile in atherosclerotic plaques containing arteries of ApoE knockout mice is profoundly different from wild type. Specifically, 852 genes were found as differentially expressed and the majority of them appear after the age of 32 weeks. The indicated altered processes, as revealed by ontology-based enrichment analysis, include adhesion and signal transduction, differentiation, apoptosis, and immune response, reflecting the cellular and molecular complexity of atherosclerosis and the cross-talk of endothelial and immune cells in aortic lesions. Cluster analysis revealed three major groups of genes with similar expression profiles, which were further analyzed, in order to find functional (GO-based) subgroups in each cluster. In agreement with the notion that atherosclerosis is an inflammatory disease [2], immune response and inflammation were the prominent categories indicated as significantly altered in the case of cluster 2, which contains genes upregulated both in 36- and 78-weeks-old mice. Promoter analysis of the genes under these categories revealed common binding elements that could contribute to a common transcriptional regulation. In particular, all of the tested genes (100%) contain cis-elements of the KLF and RXR family. The KLF family groups binding sites recognized by Krueppel like transcription factors (KLFs) [28] which are involved in many physiological and pathological processes, such as cell differentiation, proliferation, cell growth, and apoptosis during normal development or under different disease conditions. It is noteworthy that KLFs have been implicated in acute and chronic inflammatory disease states, such as atherosclerosis, diabetes, and airway inflammation [29]. It is important to note that despite the identification of KLF *cis*-acting elements, Klf4 TF was also found upregulated, suggesting that this factor could be involved in the regulation of the observed stimulation of the immune response related mechanisms. Klf4 has been found to regulate monocyte differentiation and to activate the macrophages to induce inflammation [30]. Furthermore, Klf4 regulates the proliferation and differentiation in vascular smooth muscle cells after injury to the vessel [31]. However, Klf4 seems to have an anti-inflammatory role in endothelial cells [28]. Regarding RXR family, it groups together motifs related to the receptors of retinoids, which are recognized by various heterodimers of retinoid X receptors (RXRs) and retinoic acid receptors. Interestingly, RXR has been reported to regulate several genes related to metabolic homeostasis and inflammation [32]. RXR form heterodimers with many different nuclear receptors, PPARs, LXR, and FXR affecting different aspects of cholesterol metabolism in macrophages, something known to be important in the development of atherosclerosis [32]. In addition, among the identified putative TF binding sites there are interferon regulatory factors-related elements (IRFs) in the 86% of the promoters, as well as glucocorticoid responsive elements (GREs) in the 92% of the tested promoters. In agreement, Irf8, a transcription factor involved in modulation of immune response and as a central element in the IFN signaling cascade, was found significantly overexpressed, suggesting that Irf8, together with Klf4, could be involved in the upregulation of the immune response related genes. Regarding GREs, it is well known that glucocorticoid receptors play important roles in both physiological and pathological conditions involving immunity and inflammation and that they are involved in the pathology of cardiovascular diseases [33]. Finally, Table 4 includes several genes implicated to various aspects of the disease. It is noteworthy to mention Tlr2, a member of the Toll-like receptors family, which plays a fundamental role in activation of innate immunity [34]. Furthermore, the identification of Psen2 (presenillin 2), a gene implicated in Alzheimer's disease, as candidate hub gene is interesting because genes implicated in Alzheimer's have been reported to affect cholesterol or lipoprotein function and have also been implicated in atherosclerosis [35]. 

Concluding, this bioinformatic analysis of ApoE knockout mice revealed critical altered cellular mechanisms governing atherosclerosis and indicated important molecular players that could be important targets for treatment of this complex disease.

## Supplementary Material

Supplementary Table 1: List of significantly differentiated probesets. ANOVA analysis between fold changes in expression of ApoE knockout and wild type mice, in three different ages, yielded a list of 1033 significantly differentiated probesets.
Supplementary Table 2: Kegg pathways based analysis. The list of 852 significantly altered genes was submitted to StRAnGER analysis, elucidating over-represented Kegg terms. Kegg term P value represents the hypergeometric test P value score for each Kegg term. Enrichment represents the ratio of the number of times a Kegg term occurs in the 852 gene list to the number of times this term exists in the list of the Affymetrix 430 2.0 array.

## Figures and Tables

**Table 1:** GO-analysis. The list of 852 significantly altered genes was submitted to GO analysis elucidating over-represented GO terms. GOT *P* value represents the hypergeometric test *P* value score for each GO term. Enrichment represents the ratio of the number of times a GO term occurs in the 852 gene list to the number of times this GO term exists in the list of the Affymetrix 430 2.0 array.

GO annotation	GOT *P* value	Enrichment
Ion transport	0.00000000003	33/498
Signal transduction	0.00000000004	44/803
Cell differentiation	0.00000000005	36/480
Immune response	0.00000000005	32/250
Metabolic process	0.00000000007	38/542
Cell adhesion	0.00000000011	36/387
Protein amino acid phosphorylation	0.00000000059	32/497
Multicellular organismal development	0.00000000128	41/770
Proteolysis	0.00000000690	25/358
Apoptosis	0.00000010814	24/383
Lipid metabolic process	0.00000328813	15/212
Protein transport	0.00003352383	22/465
G-protein coupled receptor signaling	0.00028681297	19/436
Oxidation reduction	0.00034119987	21/510
Cell cycle	0.00043684087	18/417
Cell division	0.00050194998	12/231

**Table 2:** Expression of genes under the GO term immune response. Values in the three last columns depict fold changes between ApoE knockout and age-matched wild type mice in log2 scale. The majority of genes at 32 and 78 weeks are upregulated.

Symbol	Description	6 weeks	32 weeks	78 weeks
Ccl6	Chemokine (C-C motif) ligand 6	−0.17	2.25	2.23
Cd74	CD74 antigen (invariant polypeptide of major histocompatibility complex, class II antigen-associated)	0.29	1.56	1.43
C1qb	Complement component 1, q subcomponent, beta polypeptide	−0.23	2.28	2.43
H2-Ab1	Histocompatibility 2, class II antigen A, beta 1	−0.25	1.94	2.77
C1qa	Complement component 1, q subcomponent, a polypeptide	−0.08	1.98	2.18
Tlr2	Toll-like receptor 2	0.14	0.99	1.38
Ccl7	Chemokine (C-C motif) ligand 7	−0.98	1.59	4.42
C4b	Complement component 4B (Childo blood group)	0.23	1.2	1.74
Cblb	Casitas B-lineage lymphoma b	0.2	0.34	1.36
Fcgr2b	Fc receptor, IgG, low affinity IIb	−0.1	1.93	2.19
Cd300lb	CD300 antigen like family member B	−0.46	2.83	2.87
Susd2	Sushi domain containing 2	0.22	−0.08	−1.07
Ccl8	Chemokine (C-C motif) ligand 8	0.33	2.92	3.01
Cd14	CD14 antigen	−0.14	1.52	2.44
Fcgr1	Fc receptor, IgG, high affinity I	−0.05	1.82	2.03
Cadm1	Cell adhesion molecule 1	−0.57	1.5	2.01
C2	Complement component 2 (within H-2S)	−0.81	1.02	−0.03
Clec7a	C-type lectin domain family 7, member a	0.15	3.52	3.86
Procr	Protein C receptor, endothelial	−0.25	0.77	1.02
C1qc	Complement component 1, q subcomponent, C chain	−0.22	2.22	2.25
Ccl19	Chemokine (C-C motif) ligand 19	−0.71	1.07	1.64
Enpp3	Ectonucleotide pyrophosphatase/phosphodiesterase 3	0.24	−0.00	−1.28
Cx3cl1	Chemokine (C-X3-C motif) ligand 1	0.18	1.71	1.87
Ccl9	Chemokine (C-C motif) ligand 9	−0.46	1.74	2.19
H2-Eb1	Histocompatibility 2, class II antigen E beta	0.19	1.62	2.17
H2-Aa	Histocompatibility 2, class II antigen A, alpha	0.95	2.39	2.34
Cxcl12	Chemokine (C-X-C motif) ligand 12	−0.34	1.1	1.95
Enpp1	Ectonucleotide pyrophosphatase/phosphodiesterase 1	0.14	0.37	1.08
Rnf19b	Ring finger protein 19B	−0.29	0.73	2.58
Prg4	Proteoglycan 4	0.16	3.33	3.99
Irf8	Interferon regulatory factor 8	0.28	1.9	2.61
Cxcl1	Chemokine (C-X-C motif) ligand 1	−0.46	1.77	2.66

**Table 3:** Common TF motif families in the promoters of 36 genes belonging to cluster 2 and to the categories “immune response” and “inflammatory response.” The percentage column depicts the percentage of genes whose promoters have at least one match with the respective motif family. Percentages and *P* value calculations are based on mouse promoters.

Family	Description	*P* value	%
V$CTCF	CTCF and BORIS gene family	0.00000185	86
V$MZF1	Myeloid zinc finger 1 factors	0.00000878	89
V$EGRF	EGR/nerve growth factor induced protein C and related factors	0.00011981	86
V$SRFF	Serum response element binding factor	0.00012387	83
V$PLAG	Pleomorphic adenoma gene	0.00015650	86
V$GREF	Glucocorticoid responsive elements	0.00016410	92
V$KLFS	Krueppel like transcription factors	0.00024104	100
V$GLIF	GLI zinc finger family	0.00170920	81
V$STAT	Signal transducer and activator of transcription	0.00177177	92
V$PAX5	PAX-2/5/8 binding sites	0.00246076	89
V$E2FF	E2F-myc activator/cell cycle regulator	0.00400401	86
V$XBBF	X-box binding factors	0.00470139	89
V$GATA	GATA binding factors	0.00499105	94
V$PAX6	PAX-4/PAX-6 paired domain binding sites	0.00504198	89
V$ETSF	Human and murine ETS1 factors	0.00646776	100
V$GCMF	Chorion-specific TFs with a GCM DNA binding domain	0.00921126	83
V$HEAT	Heat shock factors	0.01639280	92
V$RXRF	RXR heterodimer binding sites	0.01834270	97
V$FKHD	Fork head domain factors	0.02095320	94
V$IRFF	Interferon regulatory factors	0.02221250	86
V$HAND	Twist subfamily of class B bHLH transcription factors	0.03199970	94
V$ABDB	Abdominal-B type homeodomain transcription factors	0.04170260	89

**Table 4:** GOrevenge prioritization. The second column refers to the number of GO terms remaining after Gorevenge pruning, reflecting the centrality of each gene, while the third column refers to the original number of biological process category GO terms of each gene. Values in the three last columns depict fold changes between ApoE knockout and age-matched wild type mice in log2 scale. All presented genes are also differentially expressed. Top 20 genes are shown.

Gene symbol	Remaining GO terms	Original GO terms	6 weeks	32 weeks	78 weeks
Wnt5a	63	112	0.04	−0.38	−1.9
Fgfr2	56	92	0.15	−0.39	−1.05
P2rx7	38	73	0.02	0.61	1.84
Igf1	34	56	−0.23	0.77	1.39
Thbs1	30	42	−0.02	1.59	1.99
Ptgs2	30	37	−0.27	1.76	1.7
Foxf1a	28	34	0.09	−0.63	−1.41
Psen2	25	37	−0.4	0.23	1.02
Ccnd1	24	37	−0.01	0.67	1.07
Slc11a1	24	40	−0.16	1.29	1.9
Lyn	24	33	−0.01	1.07	2.26
Cebpa	24	30	−0.3	0.21	1.77
Tlr2	21	47	0.14	0.99	1.38
Osr1	21	33	0.09	0.08	−1.41
Hexb	19	23	−0.04	0.67	1
Col1a1	19	29	0.02	0.57	1.01
Socs3	19	27	0.58	1.59	3.19
Adam17	18	29	−0.22	0.27	1.07
Cd44	18	20	0.15	1.46	1.63
Cln8	18	26	0.37	0.85	1.71

**Table 5:** Prioritization of the genes presented in Table 4 by BioGraph exploiting unsupervised methodologies for the identification of causative disease-associated genes.

Gene symbol	Score
PTGS2	0.003895
CCND1	0.000566
CD44	0.000279
COL1A1	0.000194
ADAM17	0.000168
IGF1	0.000116
FGFR2	0.000116
THBS1	0.000097
LYN	0.000088
SOCS3	0.000087
CEBPA	0.000054
TLR2	0.000048
PSEN2	0.000045
P2RX7	0.000038
WNT5A	0.000035
lSLC11A1	0.000024
CLN8	0.000007
FOXF1	0.000006
HEXB	0.000005
OSR1	0.000002
